# Increased RNAi Efficiency by ds*EGFP*-Induced Up-Regulation of Two Core RNAi Pathway Genes (*OfDicer2* and *OfAgo2*) in the Asian Corn Borer (*Ostrinia furnacalis*)

**DOI:** 10.3390/insects13030274

**Published:** 2022-03-10

**Authors:** Yunhe Fan, Mureed Abbas, Xiaojian Liu, Yanli Wang, Huifang Song, Tao Li, Enbo Ma, Kun Yan Zhu, Jianzhen Zhang

**Affiliations:** 1 Institute of Applied Biology, Shanxi University, Taiyuan 030006, China; 201813102001@email.sxu.edu.cn (Y.F.); malikmureed05@gmail.com (M.A.); xiaojianliu@sxu.edu.cn (X.L.); wangyanli627631821@sxu.edu.cn (Y.W.); litao@sxu.edu.cn (T.L.); maenbo2003@sxu.edu.cn (E.M.); 2 College of Life Science, Shanxi University, Taiyuan 030006, China; 3 Modern Research Center for Traditional Chinese Medicine, The Key Laboratory of Chemical Biology and Molecular Engineering of Ministry of Education, Shanxi University, Taiyuan 030006, China; 4 Faculty of Biological Science and Technology, Changzhi University, Changzhi 046000, China; songhuifang88@128.com; 5 Department of Entomology, 123 Waters Hall, Kansas State University, Manhattan, KS 66506, USA

**Keywords:** Core RNAi pathway gene, *Ostrinia furnacalis*, RNAi efficiency, siRNA-mediated pathway

## Abstract

**Simple Summary:**

RNA interference (RNAi) has shown great potentials as a novel technology for insect pest management. However, numerous studies have shown that the efficiency of RNAi varies substantially among different insect species. For example, as a major insect pest of corn, the Asian corn borer (*Ostrinia furnacalis*) showed very low RNAi efficiency. Therefore, it is necessary to develop new strategies for enhancing RNAi efficiency in insects with low RNAi efficiency. In this study, six core RNAi pathway genes were identified and characterized from *O. furnacalis* transcriptome database. After ds*EGFP* was injected into *O. furnacalis*, the expression of the core RNAi pathway genes (*OfDicer2* and *OfAgo2*) was significantly up-regulated in response to the exposure of ds*EGFP*. As a result, the RNAi efficiency against the target genes in certain tissues of *O. furnacalis* was significantly improved. These results suggest that RNAi efficiency can be improved by inducing the expression of key RNAi pathway genes in *O. furnacalis*.

**Abstract:**

RNA interference (RNAi) is a sequence-specific gene silencing mechanism that holds great promise for effective *management* of agricultural *pests*. Previous studies have shown that the efficacy of RNAi varies among different insect species*,* which limits its wide spread application in the field of crop protection. In this study, we identified and characterized six core RNAi pathway genes including *OfDicer1*, *OfDicer2*, *OfR2D2*, *OfAgo1*, *OfAgo2,* and *OfAgo3* from the transcriptomic database of the Asian corn borer (*Ostrinia furnacalis*). Domain analysis showed that the six deduced proteins contained the necessary functional domains. Insect developmental stage- and tissue-specific expression analysis showed that five genes were expressed in all the stages and tissues examined except *OfAgo3*, which showed low expression in larvae, and high expression in pupae and adults and in the midgut. RT-qPCR was performed to examine the response of these six genes to exogenous double-stranded RNA (dsRNA). Interestingly, the transcript levels of *OfDicer2* and *OfAgo2* were significantly enhanced after the injection of ds*EGFP* at different time points and tissues investigated. Consequently, the RNAi efficiency in targeting the insect endogenous genes can be greatly enhanced in the hemolymph or midgut. Taken together, our investigations suggest that RNAi efficiency can be enhanced by pre-injection of dsRNA to induce the RNAi core machinery genes, which could be a useful strategy to improving RNAi efficiency for studying gene functions under laboratory conditions.

## 1. Introduction

RNA interference (RNAi) has become a powerful tool for gene functional studies and next-generation insect pest control [1,2], since its discovery by Andrew Z. Fire and Craig C. Mello in 1998 [3]. Previously, a myriad of reports have shown robust and systemic RNAi responses in various insect species belonging to Coleoptera [2,4] and Orthoptera [1,5,6]. However, in some insect species of Diptera, Lepidoptera, and Hemiptera, RNAi efficiency appeared very poor [7,8,9,10,11]. This variability in RNAi efficiency among various insect species greatly limits the widespread use of RNAi technology in both basic research and field applications for pest management.

There are several potential factors affecting RNAi efficiency [12]: *(1) Stability of dsRNA*. Degradation of dsRNA by nucleases (dsRNase or REase) has been documented to be the primary factor in reducing RNAi efficiency in many insects, whether in Orthoptera (susceptible to RNAi by injection) or in Lepidoptera (insusceptible to RNAi) [8,13,14,15]. Studies have shown that suppression of nuclease (dsRNase or REase) gene expression with RNAi [13,15] or application of liposomes or nanomaterials to protect dsRNA [16,17] could improve the stability of dsRNA. *(2) Cellular uptake of dsRNA*. Internalization of dsRNA into cells is an essential step to generate high RNAi efficiency. The approaches for overcoming limitations in dsRNA uptake in insects include expressing dsRNA in recombinant microbes for repeated oral delivery to insects [18], genetically engineering symbiotic bacteria to express and deliver dsRNA intracellularly [19], and enhancing clathrin-dependent endocytosis using arachidonic acid or hydrogen peroxide as was done for *B. dorsalis* [20,21]. *(3) RNAi core machinery*. Even if dsRNA is internalized and reaches the core machinery of the RNAi pathway in the cytoplasm (i.e., reaching the core RNAi enzymes), differences in the expression and function of the core RNAi enzymes could contribute to differences in RNAi efficiency among insects [22]. In *Leptinotarsa decemlineata*, expression of the core siRNA enzymes peaks in young larvae, when RNAi efficiency is the highest [23]. In contrast, a study did find a correlation between low RNAi efficiency in reproductive tissues of *Schistocerca gregaria* and low expression of *Dicer2* and *Ago2* [24].

The Asian corn borer (*Ostrinia furnalalis*) is one of the most destructive pests of corn in Asia, South-east Asia, and Oceania. In China, despite numerous control measures, this pest causes an estimated yield loss of 6–9 million tons per year. At present, its control relies mainly on synthetic insecticides and transgenic crops producing *Bacillus thuringiensis* (Bt) toxins. However, the heavy use of insecticides and Bt corn has resulted in some severe problems such as environmental pollutions and insecticide resistance in insect populations [25]. To hinder these catastrophic effects, there is an urgent need to find alternate approaches for insect pest management. In recent years, RNAi technology has emerged as a promising tool for pest management. However, RNAi has proven difficult to achieve in *O*. *furnalalis* [8]. Previously, studies have shown that degradation of dsRNA by OfREase and OfdsRNase2 is the reason for the low RNAi efficiency in *O.* *furnalalis* [8,13]. However, there has been no research on relationship between the expression levels of core RNAi pathway genes and RNAi efficiency in *O.* *furnalalis*. In this study, we identified six core RNAi pathway genes (*OfDicer1*, *OfDicer2*, *OfR2D2*, *OfAgo1*, *OfAgo2,* and *OfAgo3)* from the transcriptomic databases of *O. furnacalis* and examined the relationship between the up-regulation of these genes and the RNAi efficiency against target genes.

## 2. Materials and Methods

### 2.1. Insect Rearing

The eggs of *O. furnacalis* were purchased from Keyun, Henan Province, China, and were continuously reared in the laboratory at the Institute of Plant Protection, Shanxi Academy of Agricultural Sciences (Taiyuan, Shanxi, China) as described in Fan et al. [8].

### 2.2. Identification and Sequence Analysis of Core RNAi Pathway Genes

The candidate core RNAi pathway genes were identified in the transcriptomic databases of *O. furnacalis* through local BLAST using the sequences of *Bombyx mori* Dicer1 (XP_028040138.1) and Ago1 (NP_001095931.1), and *Ostrinia nubilali OnDicer2* (MT921812), *OnR2D2* (MT981255), *OnAgo2* (MT524717), and *Drosophila melanogaster* DmAgo3 (EF211827) as query sequences based on annotation information of cDNA sequences. The identities of the candidate genes were further confirmed using BLAST searches against the National Center for Biotechnology Information (NCBI) database (https://blast.ncbi.nlm.nih.gov, accessed on 30 November 2020. The obtained cDNAs were translated into protein sequences using the ExPASy-translational tool (https://web.expasy.org/translate/, accessed on 30 November 2020). SMART domain analysis tool (http://smart.embl-heidelberg.de/, accessed on 30 November 2020) was used to predict domain architecture.

### 2.3. Expression Analysis of Core RNAi Pathway Genes

Total RNA was isolated from the whole bodies of *O. furnacalis* at seven developmental stages (first to fifth instar larva, pupa and adult), and three tissues (hemolymph, integument, and midgut) dissected from 2-day-old fifth-instar larvae. We chose these three tissues because integument and midgut are the main exposed tissues when dsRNA is delivered by spray and feeding, respectively, whereas hemolymph is a key exposed tissue when dsRNA is delivered by injection. TRIzol reagent (Takara, Dalian, China) was used to homogenize the whole body and different tissues harvested from the larvae of *O. furnacalis*. Five biological replicates were made, and three tissues or individuals were pooled for each biological replicate. The quality and concentration of total RNA were determined using a NanoDrop 2000 spectrophotometer (Thermo Fisher Scientific, Waltham, MA, USA), and 1 μg of total RNA was taken for reverse-transcription using reagents and enzymes from Takara (Dalian, China). The synthesized cDNA samples were diluted ten-fold for use as template for reverse transcription quantitative PCR (RT-qPCR) analysis. RT-qPCR was performed using SYBR ^®^ Green Real-time PCR Master Mix (Promega, Madison, WI, USA) in a Light Cycler ^®^ 480II (Roche, Basel, Switzerland). The total volume of RT-qPCR reaction was 20 μL, containing 10 μL of 2 × SYBR mix, 0.8 μL of primers (F/R), 4 μL cDNA templates, and 4.4 μL ddH_2_O. The cycling conditions were: 94 °C for 2 min, followed by 40 cycles of 94 °C for 15 s, 60 °C for 31 s. Primers used for quantification of mRNA levels of core RNAi pathway genes were designed using primer Premier 5.0 software (Supplemental Table S1). Ribosomal protein S3 gene (*OfRpS3*, EU275206) was used as an internal control. *OfRpS3* has been proven a suitable reference gene and widely applied in RNAi research of *O. furnacalis* [26,27]. Melting curves were analyzed to confirm the specificity of amplification. Relative mRNA levels were calculated as expression of the target gene relative to the mean of the reference gene using the 2^−∆Ct^ method [28].

### 2.4. Expression of Core RNAi Pathway Genes after dsRNA Injection

Double-stranded RNA (dsRNA) was synthesized using T7 RiboMAX™ Express RNAi System (Promega, Madison, WI, USA). Primers for dsRNA synthesis were designed using the E-RNAi web service (http://www.dkfz.de/signaling/e-rnai3/, accessed on 30 January 2020). The 2× Taq Master Mix kit (Takara, Dalian, China) was used to synthesize the templates containing a T7 promoter sequence at each end. The amplified products were visualized by agarose gel electrophoresis (1%), and the PCR products were purified using the E.Z.N.A^TM^ Gel Extraction Kit (Omega Bio-Tek, Norcross, GA, USA). PCR products were used directly as templates for the synthesis of dsRNA. The synthesized dsRNA was dissolved in nuclease-free water, and the final concentration of the dsRNA was adjusted to 2.5 μg/μL.

To investigate transcriptional responses of the core RNAi pathway genes at different times after injection of dsRNA, 2-day-old fifth-instar larvae (L5D2) of *O. furnacalis* were injected with ds*EGFP* at 5 μg/larva and then placed on diets. The larvae injected with ddH_2_O served as controls. Three individuals were collected as a sample at each of four time points (2, 4, 6, and 12 h) for expression analysis with RT-qPCR.

To investigate transcriptional responses of *OfDicer2* and *OfAgo2* in different tissues after the injection of dsRNA, each of L5D2 larvae was injected with 5 μg ds*EGFP* and then placed on the artificial diet. Larvae injected with ddH_2_O were used as controls. After 2 h, hemolymph, integument, and midgut were dissected and collected in ice-cold 1.5-mL microcentrifuge tubes. Total RNA was extracted and RT-qPCR was performed to detect the expression of *OfDicer2* and *OfAgo2* as described above. Five biological replicates were made and three tissues were pooled as a biological replicate.

### 2.5. RNAi Efficiency Detection after Injection of dsRNA

The experimental design consisted of six different treatments, including ddH_2_O+ds*EGFP*, ds*EGFP*+ds*EGFP*, ddH_2_O+ds*OfEF1α*, ds*EGFP*+ds*OfEF1α*, ddH_2_O+ds*OfCTP8*, and ds*EGFP*+ds*OfCTP8*. A total of 90 L5D2 larvae of *O. furnacalis* were collected and divided into six groups with an average of 15 larvae per group. Each larva was first injected with a dose of 5 μg of ds*EGFP* (concentration: 2.5 μg/μL) or ddH_2_O. After 2 h of first injection each larva was reinjected with a dose of 10 μg (concentration: 4 μg/μL) of dsRNA. Twenty-four hours after the second injection, each larva was dissected, and hemolymph, integument, and midgut tissues were collected in ice-cold 1.5-mL microcentrifuge tubes for the extraction of total RNA. First-strand cDNA was synthesized from 1 μg of total RNA using M-MLV reverse transcriptase (Takara, Dalian, China). RT-qPCR was then performed to detect the relative expression of target genes. Specifically, the transcript levels of *OfEF1α* were determined in the treatments of ddH_2_O+ds*EGFP*, ddH_2_O+ds*OfEF1α*, ds*EGFP*+ds*EGFP,* and ds*EGFP*+ds*OfEF1α*, whereas the transcript levels of *OfCTP8* were determined in the treatments of ddH_2_O+ds*EGFP*, ddH_2_O+ds*OfCTP8*, ds*EGFP*+ds*EGFP,* and ds*EGFP*+ds*OfCTP8*.

### 2.6. Statistical Analysis

Data were analyzed using one-way ANOVA followed by Tukey’s test for multiple comparisons when the data from more than two groups were compared. However, data were analyzed with a Student’s *t*-test when the data from two groups were compared. A *p* value < 0.05 was considered statistically significant (* *p* < 0.05, ** *p* < 0.01).

## 3. Results

### 3.1. Identification and Domain Analysis of Core RNAi Pathway Genes

A total of six core RNAi pathway genes, including two *OfDicers* (*OfDicer1* and *OfDicer2*), one *OfR2D2* and three *OfAgos* (*OfAgo1*, *OfAgo2,* and *OfAgo3*), were identified through local BLAST in the transcriptomic database of *O. furnacalis* (Table 1).

**Figure 1:**
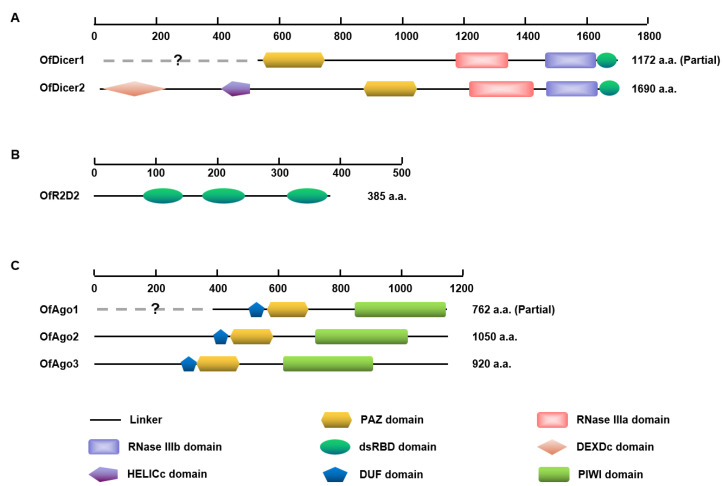
Schematic diagram of deduced domains of Dicer1, Dicer2 (**A**), R2D2 (**B**), Ago1, Ago2, and Ago3 (**C**). Blank lines represent linker regions; yellow hexagons represent PAZ domains; pink quadrilaterals represent RNase IIIa domain; purple quadrilaterals represent RNase IIIb domain; green ellipses represent dsRBD domain; pink diamond represent DEXDc domain; purple pentagon represents HELICc domain; blue pentagons represent DUF domains and green quadrilaterals represent PIWI domain.

Domain analysis showed that almost all predicted OfAgo proteins contained common domains including DUF, PAZ, and PIWI. OfDicer2 contained each of DEXDc, HELICc, PAZ, RNase IIIa, RNase IIIb, and dsRBD domains, whereas the DEXDc and HELICc domains are absent in OfDicer1, probably due to the partial sequences in the database. In contrast, OfR2D2 contained three dsRBD domains (Figure 1). The PAZ domain are specialized to bind RNA ends, especially duplex ends with short (approximately 2 nt) 3′ overhangs in Ago proteins, and the PIWI domain is unique to the Argonaute superfamily. The PAZ and RNase III domains play central roles in excising siRNAs preferentially from ends of dsRNA molecules in Dicer proteins. The function of dsRBD is to bind dsRNA [29].

### 3.2. Expression Patterns of Core RNAi Pathway Genes

**Figure 2:**
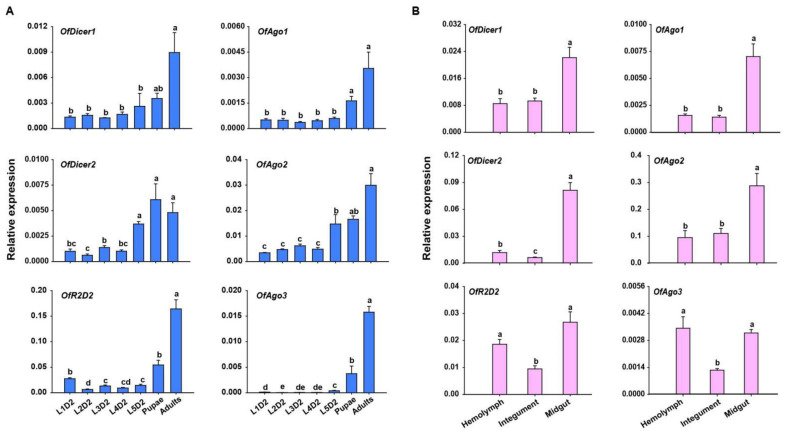
RT-qPCR analyses of relative expression of core RNAi pathway genes in different developmental stages and tissues of *O. furnacalis*. (**A**) Relative expression of *Dicer1*, *Dicer2*, *Ago1*, *Ago2*, *Ago3,* and *R2D2* at different developmental stages of *O. furnacalis*. L1D2-L5D2: Two-day-old first instar larvae to two-day-old fifth instar larvae. (**B**) Relative expression of *Dicer1*, *Dicer2*, *Ago1*, *Ago2*, *Ago3,* and *R2D2* in different tissues including hemolymph (HE), integument (IN), and midgut (MG) in two-day-old fifth-instar larvae. Statistical significance was determined using a one-way ANOVA followed by Tukey’s post hoc test. Different letters (a–e) above the bars represent significant differences (n = 5) (*p* < 0.05).

To examine the transcription levels of core RNAi pathway genes, RT-qPCR was performed in seven developmental stages of *O. furnacalis*. The expression profiles showed that almost all the candidate genes were expressed in all the developmental stages examined except for *OfAgo3* which showed the expression only in pupal and adult stages, but not in the larval stage. Generally, the expression of almost all the candidate genes was lowest in the larval stage, and gradually increased in the rest of developmental stages. Four candidate genes, including *OfDicer1*, *OfDicer2*, *OfAgo1,* and *OfAgo2*, displayed higher expression in pupal stage. However, almost all the candidate genes showed the highest expression in the adult stage (Figure 2A).

Tissue-specific expression profiles in L5D2 larvae showed that core RNAi pathway genes were expressed in all tissues investigated. *OfAgo1*, *OfAgo2*, *OfDicer1,* and *OfDicer2* were highly expressed in the midgut, whereas *OfAgo3* and *OfR2D2* displayed highest expression in the hemolymph and midgut (Figure 2B).

### 3.3. Transcriptional Responses of Core RNAi Pathway Genes to Injection of dsEGFP

**Figure 3:**
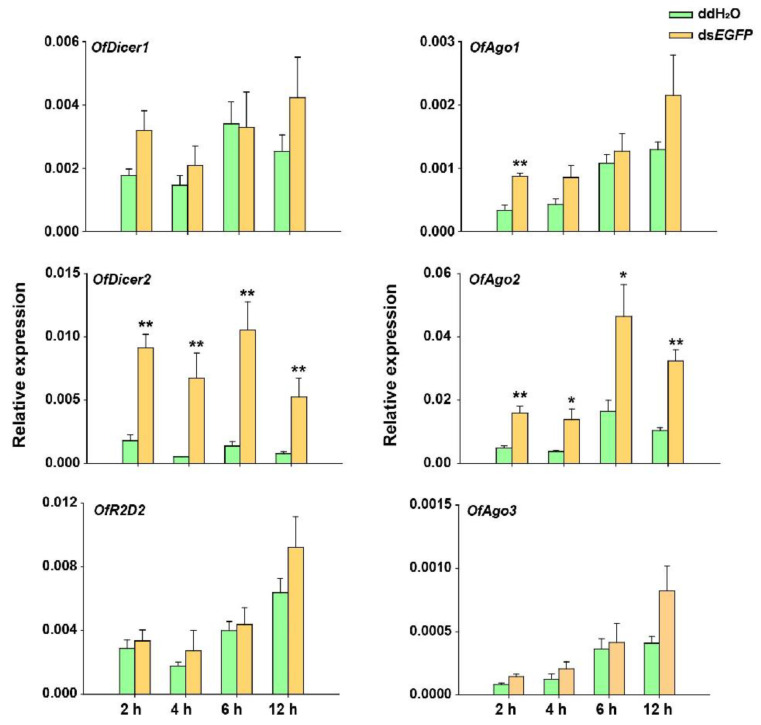
RT-qPCR analyses of relative expression of *Dicer1*, *Dicer2*, *Ago1*, *Ago2*, *Ago3,* and *R2D2* in *O. furnacalis* at different time-points after each larva was injected with 5 μg ds*EGFP*. ddH_2_O was injected in the negative control. Each treatment consisted of five replicates and three individuals were pooled in each replicate. Student’s *t*-test was used in statistical analysis of the data (*, *p* < 0.05, **, *p* < 0.01).

After introduction of ds*EGFP* into *O. furnacalis,* the response of RNAi pathway genes was investigated under two different experimental conditions. In the first experiment, larvae injected with 5 μg of ds*EGFP* in each were collected at 2, 4, 6, and 12 h and the expression levels of the respective core RNAi pathway genes were evaluated using RT-qPCR. The results demonstrated that there was no significant change in the relative expression of *OfAgo3*, *OfDicer2,* and *OfR2D2* at any of these time points. However, *OfAgo1* was up-regulated only at 2 h after dsRNA injection. To our surprise, the expression levels of *OfAgo2* and *OfDicer2* were significantly increased after the injection of ds*EGFP* compared with the injection of ddH_2_O as control at each time interval (Figure 3).

**Figure 4:**
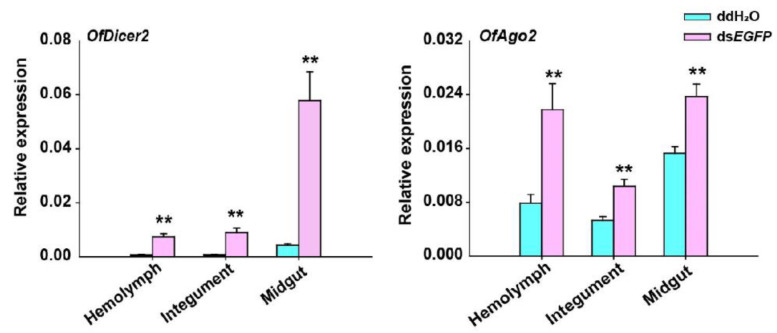
RT-qPCR analyses of relative expression of *Dicer2* and *Ago2* in different tissues including hemolymph (HE), integument (IN), and midgut (MG) 2 h after injection of ds*EGFP*. ddH_2_O was injected in the negative control. Each treatment contained five replicates and three individuals were pooled in each replicate. Student’s *t*-test was used in statistical analysis of the data (**, *p* < 0.01).

Since the expression of *OfDicer2* and *OfAgo2* was up-regulated for a long time after injection of ds*EGFP*, we evaluated the expression levels of *OfDicer2* and *OfAgo2* in three different tissues including hemolymph, integument, and midgut by RT-qPCR. Our results showed that the expression level for *OfDicer2* in the hemolymph, integument, and midgut increased by 9.7-, 11.0-, and 13.5-fold, respectively, whereas the expression level of *OfAgo2* increased in the hemolymph, integument, and midgut by 2.8-, 2.0-, and 1.5-fold, respectively (Figure 4).

### 3.4. RNAi Efficiency Can Be Improved by Pre-Injection of dsEGFP

**Figure 5:**
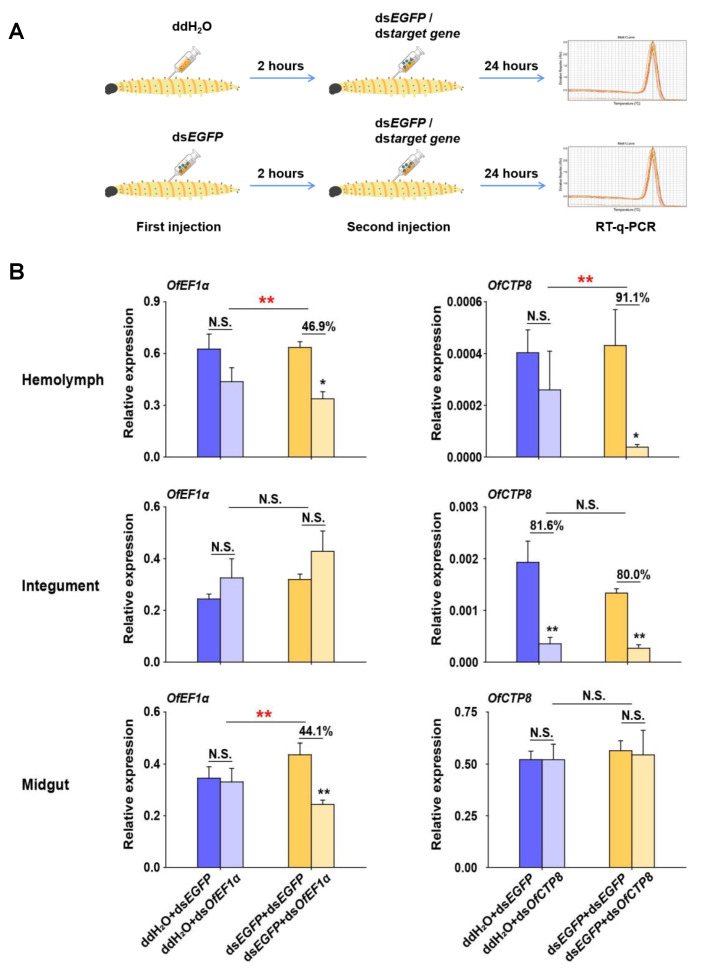
Enhanced RNAi efficiency by pre-injection of ds*EGFP* in different tissues of *O. furnacalis*. (**A**) Overview of the experimental setup. ddH_2_O or ds*EGFP* was pre-injected into larvae (L5D2). After 2 h, ds*EGFP* or ds*OfEF1α/dsOfCTP8* was injected into the larvae again. After 24 more hours, RT-qPCR was performed to examine the expression of *OfEF1α* or *OfCTP8* in different tissues of *O. furnacalis*. (**B**) Different combinations of dsRNA or ddH_2_O injection were performed for this assay. The statistical significance between control and treatment results was assessed by Student’s *t*-test. Bars show mean ± SE (* *p* < 0.05; ** *p* < 0.01; N.S., nonsignificant) (n = 5).

To ascertain whether an increase in the transcript levels of *OfDicer2* and *OfAgo2* could lead to an increase in the RNAi efficiency, two target genes, *OfEF1α* (elongation factor 1*α*) and *OfCTP8* (chymotrypsins 8) were selected as marker genes. In the control group, L5D2 larvae were first injected with ddH_2_O. After 2 h, ds*EGFP* or dsRNA of each of the target genes was injected into the larvae. Twenty-four hours later, RT-qPCR was carried out to analyze the relative expression levels of the target genes. Our results demonstrated that the transcript levels for *OfEF1α* remained constant and no significant change was observed in the hemolymph, integument, and midgut, while the expression level for *OfCTP8* was efficiently silenced (81.6%) only in the integument, but not in the hemolymph and the midgut. When L5D2 larvae in the treatment group were injected with ds*EGFP* and then with the dsRNA of the target genes, the transcript level of *OfEF1α* was suppressed by 46.9% and 44.1% in the hemolymph and the midgut, respectively. In contrast, the transcript level for *OfCTP8* was suppressed by 91.9% and 80.0% in the hemolymph and the integument, respectively (Figure 5). These results suggest that RNAi efficiency can be improved in some tissues of *O. furnacalis* by pre-injecting ds*EGFP* to induce the expression of RNAi core machinery genes.

## 4. Discussion

RNAi is a highly conserved, post-transcriptional gene-silencing mechanism in which small RNA molecules are utilized by RNAi core machinery that involves several core RNAi pathway genes to degrade complementary RNA molecules in a sequence-specific nature [12]. Core RNAi pathway genes are critical to RNAi efficiency. A previous study conducted by Tomoyasu et al. [30] has shown that two *Ago2* (*TcAgo2a* and *TcAgo2b*) genes identified from the *T. castaneum* genome are involved in systemic RNAi response of RNAi. Similarly, both *LmAgo2a* and *LmAgo2b* have been found to contribute to the robust RNAi response in *L. migratoria* [31]. In *L. decemlineata,* two *Dicer2* genes (*Dicer2a* and *Dicer2b*) and two *Ago2* genes (*Ago2a* and *Ago2b*) were identified in *L. decemlineata* transcriptome database [23], which support the hypothesis that the number of core RNAi pathway genes may contribute to the variability of RNAi efficiency in insects. However, in the present study, only a single transcript of *OfDicer2* and *OfAgo2* was identified in *O. furnacalis.* It is speculated that this may be one of the reasons for the low RNAi efficiency in *O. furnacalis.*

In the present study, our results showed that the six core RNAi pathway genes (*OfDicer1*, *OfDicer2*, *OfR2D2*, *OfAgo1*, *OfAgo2,* and *OfAgo3*) were expressed in almost all the developmental stages and different tissues examined. In particular, the highest transcript level for the core RNAi pathway genes was observed at pupae and adult stages and in the midgut. Our results are in agreement with Cooper et al. [28] and Xie et al. [32] which showed that the expression patterns for *Dicer2*, *R2D2*, and *Ago2* in *O. nubilalis* and *B. dorsalis.* Nevertheless, little has been known about differential expressions of core RNAi pathway genes during different developmental stages and in different tissues in relation to RNAi efficiency in insects. Because induced expression of core RNAi pathway genes can increase RNAi efficiency as we showed in this study, we hypothesize that RNAi-mediated suppression of a target gene is relatively easy to achieve when and where the core RNAi pathway genes are highly expressed. This information provides the fundamental basis to investigate the mechanism of RNA interference in various developmental stages and tissues of *O. furnacalis*.

Many previous studies have reported that RNAi acts as an RNA-based immune system against viral infections in insects, and plays a crucial role in regulating endogenous genes when insects are infected with a virus. To combat the viral infection, the RNAi machinery’s activity can enhance gradually by up-regulating some core RNAi components such as *Dicer2* and *Ago2* [32,33,34]. Similarly, non-specific dsRNAs have also been found in implicating as a trigger to induce the RNAi activity by up-regulating the expression of core RNAi pathway genes in certain insect species [23,35,36]. For example, it was found that ds*LdSAHase*-induced suppression of *LdSAHase* and the associated larval mortalities were influenced by pre-exposure to ds*EGFP* in *Leptinotarsa decemlineata* [23]. Similarly, pre-exposure of *Acyrthosiphon pisum* to ds*GFP* (600 ng) led to significant silencing of *hunchback* gene when the aphid was exposed to 60 ng of ds*hunchback*, a dose which cannot lead to the suppression of *hunchback* expression without pre-exposure of the aphid to ds*GFP* [35]. 

In this study, we first investigated the possible association between RNAi efficiency and the expression levels of core RNAi pathway genes in *O. furnacalis*. Our result showed the mRNA levels of *OfDicer2* and *OfAgo2* were high at all of the insect developmental stages, particularly in the late larval (L5D2) stage, pupal and adult stages, and as well as in the tissues, particularly the midgut. Because *Dicer2* and *Ago2* are believed to be involved in the siRNA pathway as shown in *Drosophila* [37], high expression of *OfDicer2* and *OfAgo2* may be related to RNAi efficiency during these stages. Indeed, several studies have shown that sufficient silencing efficiency can be achieved during the wandering or pupal stages of lepidopterans, such as *B. mori* and *Spodoptera litura* [38,39], which may be related to high expression of *Dicer2* and *Ago2* during these stages. Similarly, high expression of the core RNAi pathway genes in certain tissues, particularly in the midgut, may also imply strong RNAi machinery in these tissues. However, up-regulation of *OfAgo1* was observed 2 h post-injection of ds*EGFP*, which implies that *OfAgo1* could also be involved in siRNA pathway at an early stage of dsRNA exposure in *O. furnacalis.* These results are similar to the functions of *Ago1* in *L. migratoria,* which has been shown to be involved in siRNA-mediated RNAi pathway [31]. However, further research is needed to clarify the role of *OfAgo1* in siRNA-mediated RNAi pathway. 

Nevertheless, our major focus of this study was on possible induced effects caused by injection of exogenous dsRNA (i.e., ds*EGFP*) on RNAi efficiency for attacking target genes in *O. furnacalis*. Since injection of ds*EGFP* could induce a strong response of *OfDicer2* and *OfAgo2*, *OfEF1α* and *OfCTP8* were selected as two target genes to determine whether the silencing efficiency against the two target genes could be improved by pre-injection of ds*EGFP*. Previous studies have shown that *OfEF1α* can be significantly silenced by tissue culture RNAi in *O. furnacalis* [40], and *OfCTP8* can be significantly knocked down by injection of ds*OfCTP8* in *O. furnacalis* [13]. As expected, *OfEF1α* could not be significantly silenced in any tissues (hemolymph, integument, and midgut) when *O. furnacalis* larvae were pre-injected with ddH_2_O. However, *OfEF1α* can be significantly silenced in the hemolymph and midgut, but not in the integument, when *O. furnacalis* larvae were pre-injected with ds*EGFP*. Furthermore, RNAi efficiency targeting *OfCTP8* could be significantly improved in the hemolymph by pre-injection of ds*EGFP* compared with pre-injection of ddH_2_O. Although our results are highly encouraging for improving RNAi efficiency by inducing the activity of the core machinery of the siRNA-mediated RNAi pathway through the pre-injection of ds*EGFP*, the level of the improvement for different target genes and in different tissues still varies considerably. Presumably, many other factors may contribute to such variations, including dsRNA sequence difference, different efficiencies in dsRNA uptake in different tissues, and different intensities of responses of the RNAi core machinery genes (i.e., *OfDicer2* and *OfAgo2*) to the injection of ds*EGFP* in *O. furnacalis*. Therefore, further study is needed to pinpoint the exact mechanisms causing such variations. 

## 5. Conclusions

In the current study, we identified six core RNAi pathway genes (*OfDicer1*, *OfDicer2*, *OfAgo1*, *OfAgo2*, *OfAgo3,* and *OfR2D2*) in *O. furnacalis*. Expression profiles indicated that five genes were expressed in all the developmental stages and tissues examined except *OfAgo3*, which showed low expression in larvae. However, only *OfDicer2* and *OfAgo2* were upregulated in response to ds*EGFP* exposure at all four time points and tissues investigated. In addition, the RNAi efficiency of target genes can be significantly enhanced in the hemolymph or midgut by pre-injection of ds*EGFP*. Thus, this study provides new insights into developing useful strategies for improving RNAi efficiency in RNAi-refractory insect species, particularly in laboratory studies, where pre-exposure to an exogenous dsRNA in insects is feasible.

## Figures and Tables

**Table 1:** Characteristics of core RNAi pathway transcripts and their deduced proteins from *O. furnacalis*.

	Full Length or Not	GenBank Accession Number	ORF (bp)	Protein (aa)	Mass (kDa)	Isoelectric Point (pI)
OfAgo1	no	--	2287	762	86.55	9.48
OfAgo2	yes	XP_028167628	3153	1050	117.56	9.47
OfAgo3	yes	XM_028315439	2763	920	103.93	9.12
OfDicer1	no	XP_028160127	3519	1172	132.57	5.06
OfDicer2	yes	XM_028314437	5073	1690	193.19	6.51
OfR2D2	yes	MT981255	1158	385	42.49	9.01

## Data Availability

Sequence data are available in a publicly accessible repository: GenBank accession numbers are shown in Table 1.

## Supplementary Materials

The following supporting information can be downloaded at: https://www.mdpi.com/article/10.3390/insects13030274/s1, Table S1: Primers for PCR amplification, dsRNA synthesis and RT-qPCR analysis.
