# Supplementary material for: Increased RNAi Efficiency by dsEGFP-Induced Up-Regulation of Two Core RNAi Pathway Genes (OfDicer2 and OfAgo2) in the Asian Corn Borer (Ostrinia furnacalis)
Source: Insects. 2022 Mar 10;13(3):274. doi: 10.3390/insects13030274 (PMC8948962; doi:10.3390/insects13030274)
Supplement: Supplementary file 1 [file insects-13-00274-s001.zip › insects-1604910-supplementary.pdf]

Supplemental Information for Fan et al., Up-Regulation of Core RNAi Pathway Genes

Table S1. Primers for PCR amplification, dsRNA synthesis and RT-qPCR analysis

Application of primers	Gene name	Sequence of primers (5′ -3′)	Product size (bp)
cDNA sequencing	*OfAgo1*	F: AGTGGGTGGCTCAGGTGTCA<br>R: GAAGTCGAACTCGGTGGGGT	1862
	OfAgo2	F: GAGGAAAGGAAAGCTGCTCTGA<br>R: AAGCCTGTTTCATTACTTACTGAT	4538
	OfAgo3	F: GACGTGATAATGGCGGATCCTA<br>R: TAACTCACAATCAACTTATTTCGT	2866
	OfDicer1	F: CTGTTGGATGTGGACCATACGA<br>R: GTCCCTTTAGCAATGCGATA	3056
	OfDicer2	F: CAGTCCTAAGTATTATAACTCGCC<br>R: TGCATGTGACACGCAAAATTAG	4389
	OfR2D2	F: CTGTGTAGATAGGTGCTCATT<br>R: TAAATACTGTTAGACAGCATAA	1575
dsRNA synthesis	*EGFP*	F: taatacgactcactataggggacgTAAACGGCCACAAGTT<br>R: taatacgactcactatagggTGTTCTGCTGGTAGTGGTCG	496
	OfAgo1	F: taatacgactcactatagggAAGAGGCATTAGAAGGGCGT<br>R: taatacgactcactatagggTGCAGTGGAAATGATTGCAT	460
	OfAgo2	F: taatacgactcactatagggCAAACTGCCCGCAATATTCT<br>R: taatacgactcactatagggCAGTCTCGTGTGATGTCGCT	480
	OfAgo3	F: taatacgactcactatagggTGGGGCTCCTACATTCAGTC<br>R: taatacgactcactatagggACCAAGTTCATGGCCTCATC	496
	OfDicer1	F: taatacgactcactatagggCATCTGTCACCCACCACAAG<br>R: taatacgactcactatagggTCCAAATCGGGCTGATAGTC	478
	OfDicer2	F: taatacgactcactatagggCGTGCTGTGGTACAAGGCTA<br>R: taatacgactcactatagggTAATATAGCCGCGGAAGTGG	496
	OfR2D2	F: taatacgactcactatagggGGAGATGGAGTCGTTACCCA<br>R: taatacgactcactatagggCCTGACGCTTAGCCAGTTTC	500
	OfEf1α	F: taatacgactcactatagggCCGGTACCATCGTCGTATTC<br>R: taatacgactcactatagggACGGTCTGTCTCATGTCACG	480
	OfCTP8	F: taatacgactcactatagggACTGTAGTCGAAATGCGTCT<br>R: taatacgactcactatagggAAACTCCGAAGGGCAGATAG	496
RT-qPCR analysis	*OfAgo1*	F: GGTCAGGAACACAAGCACACAT<br>R: GGTTGTTGATTTCACGCTCTCT	154
	OfAgo2	F: CCCAGAACAAACAACTGAACGATT<br>R: ACTGGTGATGACATCCTGGATCTT	104
	OfAgo3	F: TTGGAAACGGGCTATGAACG<br>R: TCACCTCTCCCTTTTCATTTTT	140
	OfDicer1	F: GTTTCCAACTTGAACCTCTACC<br>R: AGGTTGTGTATGTGTGAGAGGC	132
	OfDicer2	F: GACAAGGTGCCTCCCTACATCA<br>R: TGATGTAGGGAGGCACCTTGTC	228
	OfR2D2	F: AGGACAACCCGCCAGAGT<br>R: TGTGAAACTGCGACACCTTG	128
	OfEf1α	F: GGGTTCCTTCAAATATGCCTGG<br>R: GCCTGAGAGGTTCCTGTGAT	174
	OfCTP8	F: ATCCAGCCAATCGCGCTGCC<br>R: GCTGACCACCGAGGATGCGC	123
	OfRpS3	F: GCGTTTCAACATCCCTGAAC<br>R: CACACCATAGCAGGCACGA	143
